# Picking Your Brains: Where and How Neuroscience Tools Can Enhance Marketing Research

**DOI:** 10.3389/fnins.2020.577666

**Published:** 2020-12-03

**Authors:** Letizia Alvino, Luigi Pavone, Abhishta Abhishta, Henry Robben

**Affiliations:** ^1^Center for Marketing and Supply Chain Management, Nyenrode Business University, Breuklen, Netherlands; ^2^Neuromed, Mediterranean Neurological Institute, Isernia, Italy; ^3^Hightech Business and Entrepreneurship Group (HBE), University of Twente, Enschede, Netherlands

**Keywords:** neuromarketing, consumer neuroscience, review, iMotion, GRAIL, marketing, neurophysiological tools, physiological tools

## Abstract

The use of neuroscience tools to study consumer behavior and the decision making process in marketing has improved our understanding of cognitive, neuronal, and emotional mechanisms related to marketing-relevant behavior. However, knowledge about neuroscience tools that are used in consumer neuroscience research is scattered. In this article, we present the results of a literature review that aims to provide an overview of the available consumer neuroscience tools and classifies them according to their characteristics. We analyse a total of 219 full-texts in the area of consumer neuroscience. Our findings suggest that there are seven tools that are currently used in consumer neuroscience research. In particular, electroencephalography (EEG) and eye tracking (ET) are the most commonly used tools in the field. We also find that consumer neuroscience tools are used to study consumer preferences and behaviors in different marketing domains such as advertising, branding, online experience, pricing, product development and product experience. Finally, we identify two ready-to-use platforms, namely iMotions and GRAIL that can help in integrating the measurements of different consumer neuroscience tools simultaneously. Measuring brain activity and physiological responses on a common platform could help by (1) reducing time and costs for experiments and (2) linking cognitive and emotional aspects with neuronal processes. Overall, this article provides relevant input in setting directions for future research and for business applications in consumer neuroscience. We hope that this study will provide help to researchers and practitioners in identifying available, non-invasive and useful tools to study consumer behavior.

## 1. Introduction

Concepts, methodologies and tools in marketing have remained unchanged for a relatively long period. However, changing market structures (e.g., offline to online, globalization, hyper-competitive environment, increasing demand) demand new marketing methodologies and tools that are able to adapt to this new situation (Hackley, 2009; Armstrong et al., 2018). Thus, academics and practitioners have investigated how marketing research could benefit from the integration of methods and tools from other disciplines. In the early 2000s, a novel approach for studying consumer behavior emerged. This new approach is now known as Consumer Neuroscience (a.k.a. Neuromarketing) and lies at the intersection of three disciplines: marketing, psychology, and neuroscience (Plassmann et al., 2012).

The goal of consumer neuroscience is the study of neuropsychological mechanisms that support and lead consumer decision making and behavior. Consumer neuroscience uses both psychological and neuroscience methods to investigate marketing related issues concerning buying behavior, thus offering scientific explanation on consumer's preferences and behaviors (Levallois et al., 2012; Russo et al., 2015). There are multiple consumer neuroscience tools that are used to study consumer decision-making and behavior. Usually, consumer neuroscience tools include devices that can measure vital physiological functions (e.g., heartbeat, respiration rate, blood pressure) and reflexes (e.g., gaze fixation, pupil dilatation, face expression) (Global Harmonization Task Force, 2012). These tools reveal information about impressions, reactions (e.g., positive, negative) and emotional responses (e.g., positive, negative) when exposed to marketing stimuli (Hamelin et al., 2017). Consumer neuroscience tools also allow real-time measurements of brain activity, such as functional magnetic resonance imaging (fMRI) and electroencephalogram (EEG). These tools measure the neural activity of consumers while they perform consumption-related behavior (e.g., buying or testing a product), or in the periods directly preceding and following such behaviors (Plassmann et al., 2015; Montazeribarforoushi et al., 2017).

Many studies have focussed on the benefits of neuroscience tools in marketing (Vecchiato et al., 2011; Bercea, 2013; Hsu and Yoon, 2015; Ramsøy, 2015; Boz et al., 2017; Lee et al., 2017; Alvino, 2019; Songsamoe et al., 2019). Several studies also provide an overview of the most common neuroscience tools that could be used in consumer neuroscience tools, for instance EEG and fMRI. However, there is lack of literature that surveys these tools to provide guidance for practitioners and researchers. The aim of this article is *to provide an overview of the use and characteristics of neuroscience tools employed for studying consumer behavior*.

In order to achieve our aim we make use of a literature review as explained in section 2. After selecting relevant publications we first study the classification criteria used to categorize consumer neuroscience tools and propose criteria to classify those practically used tools in section 3. We then discuss the various characteristics of the consumer neuroscience tools in section 4. Thereafter, in section 5, we study the various applications of consumer neuroscience tools in marketing. Section 6 describes the benefits and potential of two novel ready-to-use platforms that integrate different consumer neuroscience tools together. Finally, we present the conclusions of our study in section 7.

## 2. Method

We use a literature review methodology (Webster and Watson, 2002) to survey the scientific contributions and construct an overview of the use and characteristics of neuroscience tools used to study consumer behavior. For the literature review, we have considered academic articles indexed by Scopus published between 2004 and 2019, as the first “neuromarketing” article was published in 2004 (Brammer, 2004). The first paper that uses the term “consumer neuroscience” was published in 2008 (Hubert and Kenning, 2008).

**Figure 1:**
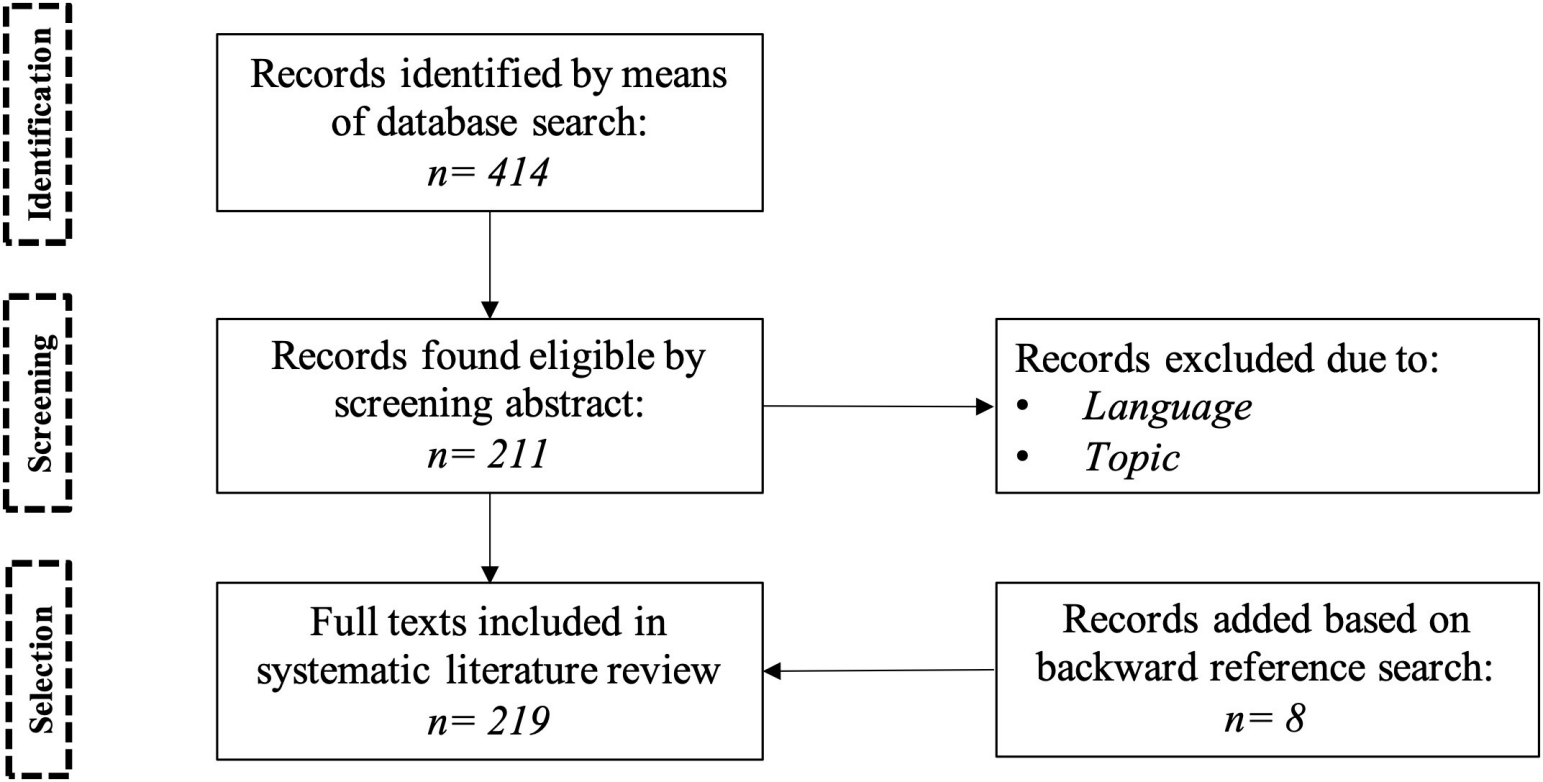
Overview of review methodology.

As shown in Figure 1, we follow a three-step process to select the studies for this review. First, we search academic records on Scopus, using the following query, *(“neuromarketing” OR (“consumer” AND “neuroscience”))*. This query returns all the records that mention “neuromarketing” or “consumer neuroscience” as a keyword. Due to a high number of records (412 records) found, we decide to screen the studies by evaluating their abstract and conclusions. We screen the records based on the following parameters:

1. We remove the records that did not focus on neuromarketing or consumer neuroscience as a research topic.
2. We only consider records in the English language.
3. We exclude the studies that were not marketing related.

Post screening a total of 211 studies were found eligible for this survey. Finally, we add 8 more records to the eligible studies based on suitable studies (including books and reports) found as a result of backward reference search. We select 219 studies that belong to several domains where consumer neuroscience is a research topic, for example, neuroscience, marketing, psychology, economics and engineering in this survey. We further evaluate the studies and group them according to the following three categories: review, empirical (based on experiments) and conceptual (based on interviews). In the selected corpus we find 137 empirical research, 69 review, 9 conceptual papers, and 4 reports.

**Figure 2:**
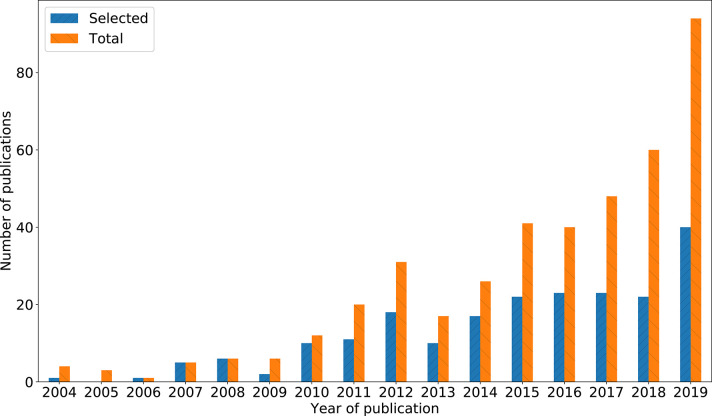
Number of publications selected for this study.

Figure 2 shows the year of publication of the selected studies. We recorded the tools discussed by each study and the tools employed for data collection in each empirical study. We recorded the various characteristics (e.g., advantages, disadvantages, cost of procurement, etc.) of the tools mentioned in the studies. In addition, we recorded the classification criteria used by the studies to categorize the tools, the marketing domain of application and the type of product or service tested. Based on the review of selected studies, we develop insights in order to achieve the objective of this review.

## 3. An Overview of Consumer Neuroscience Tools

Consumer neuroscience uses neuroscience tools to study behavior of consumers and their decision-making processes. Traditional marketing techniques such as self-reports or interviews mainly allow the measurement of conscious reactions to marketing-related stimuli (e.g., advertisements, brands). Conversely, neuroscience tools enable researchers to measure physiological signals aroused by marketing stimuli such as music, videos, brand logo, or websites (Schneider and Woolgar, 2015; Alvino et al., 2018; Alvino, 2019; Muñoz-Leiva et al., 2019).

These tools also help to understand how a consumer experiences marketing stimuli and to identify the factors that influence and modify consumers' preferences. There are several definitions and descriptions of consumer neuroscience tools (Fugate, 2008; Fisher et al., 2010; Morin, 2011; Gang et al., 2012; Venkatraman et al., 2015; Chapman et al., 2019; Lajante and Ladhari, 2019; Tobon et al., 2020). In this section, we focus on classifications of neuroscience tools as classification criteria that identify, group, and properly name tools via a standardized system.

### 3.1. Classification of Tools

Consumer Neuroscience tools are usually categorized based on the type of measurements. Studies (Kenning et al., 2007a; Boz et al., 2017; Stasi et al., 2018) have broadly divided these tools into two categories namely *physiological tools and neurophysiological tools*. Physiological tools can measure voluntary and involuntary reflexes such as fixating and tracking visual stimuli or movements of the mimetic musculature of the face (facial expressions) (Global Harmonization Task Force, 2012). Physiological tools (or methods) include electrocardiogram (ECG), electrodermal activity (EDA), participants' facial muscles fEMG, eye-tracker (ET) and voice pitch analysis (VOPAN) (Isabella et al., 2015; Boz et al., 2017; Stasi et al., 2018). Neurophysiological tools (or methods) measure and record brain activity to study consumer behavior (Boz et al., 2017). Examples of neurophysiological tools are electroencephalography (EEG), positron emission tomography (PET), magnetoencephalogaphy (MEG), functional magnetic resonance imaging (fMRI) and transcranial magnetic stimulation (TMS) (Kenning et al., 2007a,b).

Literature (Wang and Minor, 2008; Bercea, 2013; Fortunato et al., 2014; Harris et al., 2018; Lim, 2018) shows that these tools can also be classified based on the *type of brain activity* they measure; i.e., tools that:

1. Measure the metabolic activity in the brain
2. Measure electrical activity in the brain
3. Do not measure brain activity.

Wang and Minor (2008) identifies three tools that measure changes in chemical composition or changes in the flow of fluids in the brain (brain imaging analysis), namely functional magnetic resonance imaging (fMRI), positron emission tomography (PET), transcranial magnetic stimulation (TMS) or magnetoencephalogaphy (MEG). In contrast, electroencephalography (EEG) and steady-state topography (SST) measure non-hemispheric brain wave analysis and hemispheric lateralization (brain wave analysis). Bercea (2013) states that PET and fMRI can record metabolic brain activity and that EEG, SST, TMS, MEG and functional near-infrared spectroscopy (fNIRS) can record electrical brain activity. According to Harris et al. (2018), changes in metabolic brain processes are measured by fMRI, PET and functional transcranial Doppler sonography (fTCS). In contrast, changes in electrical activity are measured by various techniques including EEG, MEG, SST, and TMS. The tools that do not record brain activity can be considered physiological tools such as galvanic skin response (GSR), electrocardiogram (ECG), eye tracking (ET), facial expression recognition software (fERS), voice pitch analysis, and implicit association tests (IAT) (Wang and Minor, 2008; Bercea, 2013; Fortunato et al., 2014; Harris et al., 2018).

Similarly, Lim (2018) classifies consumer neuroscience tools into three categories, i.e., tools that:

1. Record outside brain activity
2. Record inside brain activity
3. Manipulate neural activity.

In the first category, we find tools such as GSR, ECG, ET, and fERS. The second category (tools that record neural activity) is then divided into two categories: electromagnetic and metabolic. PET and fMRI can record metabolic brain activity, and EEG, MEG, SST can record electrical brain activity (Lim, 2018). In the last category (manipulate neural activity), we find transcranial magnetic stimulation (TMS) and neurotransmitters (NTs). Neurotransmitters are chemical substances that enable the transmission of neurological signals from one neuron to another target neuron (Lim, 2018).

Finally, Ramsøy (2015) identifies four different types of consumer neuroscience tools, namely:

1. Self-reports
2. Behavioral measurement
3. Physiological measurement
4. Neuroimaging.

Self-report is one of the most widely used methods of collecting information regarding individual health status, feelings, attitudes, and beliefs. For example, it might be important to know how participants feel while shopping in a store or while performing a task (Paulhus and Vazire, 2007). Behavioral measurements reveal information about consumer behaviors, impressions, and concern particular mental states or responses (Ramsøy, 2015). In behavioral measurements, people are observed and recorded when they perform a task, for instance, reaction time (RT), which is the opposite of self-report. Physiological measurements are useful to evaluate people's biological responses to stimuli. Physiological measurements are usually not under a consumer's voluntary control, therefore they cannot be easily influenced, in contrast to self-reports and behavioral measurements (Ramsøy, 2015). Examples of physiological measurements are body language, facial expression, eye movement and pupil dilatation, palm sweating, respiration and pulse. Finally, neuroimaging refers to different tools that are used to identify and analyse brain activity. Ramsøy (2015) classifies as neuroimaging tools EEG, fMRI, MRI, PET MEG, and single photon emission tomography (SPECT).

We identify three classification criteria used in the literature for categorizing neuroscience tools. There are some similarities between some of the classifications, for instance, (Isabella et al., 2015) with (Boz et al., 2017) or (Wang and Minor, 2008) with (Bercea, 2013) and (Harris et al., 2018); we observe that those authors chose different criteria to group consumer neuroscience tools. As shown in Table 1, previous studies group consumer neuroscience tools in different number of levels (2, 3, or 4) on the base of (1) type of measurements (e.g., behavioral, physiological, neurophysiological), (2) type of neuronal activity (neuronal activity outside or inside the brain), (3) brain activity recorded (e.g., metabolic or electric), (4) no brain activity, or (5) manipulate neuronal activity. Surprisingly, some of these classifications also describe these tools erroneously. While some authors classify them as data collection methods (and not tools), some other authors mistake the tools for measurements. E.g., some authors describe eye tracking, heart rate and electrodermal activity (skin conductance) as tools, instead of properties of the human body or, more precisely, changes in eye movements, muscle contraction of the heart and electrical properties of the skin (Wiles and Cornwell, 1991; Braithwaite et al., 2013; Ramsøy, 2015). Some authors also refer to the Implicit Association Test as a tool, however, the IAT is a test that can be used to measure the strength of differential association of two or more target concepts with an attribute (Greenwald et al., 1998; Ramsøy, 2015). Finally, Lim (2018) consider neurotransmitters (NT) as consumer neuroscience methods. However, NTs are chemical substances in our brain and cannot be considered a method or a tool.

**Table 1:** Classification of consumer neuroscience tools.

**Number of levels**		**3**		**3**		**2**		**4**		**2**		**3**		**3**
Electroencephalography	**(Wang and Minor, 2008)**	BWA	**(Bercea, 2013)**	EBAc	**(Isabella et al., 2015)**	NPy	**(Ramsøy, 2015)**	NIm	**(Harris et al., 2018)**	NPy	**(Boz et al., 2017)**	EBAc	**(Lim, 2018)**	NAc (Inside)
Functional magnetic resonance imaging		BIA		MBAc		NPy		NIm		NPy		MBAc		NAc (Inside)
Functional near-infrared spectroscopy		-		EBAc		-		NIm		-		-		-
Functional transcranial Doppler sonography		-		-		-		-		-		MBAc		-
Magnetoencephalography		BIA		EBAc		NPy		NIm		NPy		EBAc		NAc (Inside)
Positron emission tomography		BIA		MBAc		NPy		NIm		NPy		MBAc		NAc (Inside)
Steady-state topography		BWA		EBAc		-		NIm		-		EBAc		NAc (Inside)
Single photon emission tomography		-		-		-		NIm		-		-		-
Transcranial magnetic stimulation		-		EBAc		NPy		-		-		EBAc		MNAc
Neurotransmitters		-		-		-		-		-		-		MNAc
Eye Tracker		NBAc		NBAc		Py		Py		Py		Py		NAc (Outside)
Electrocardiogram		NBAc		NBAc		Py		Py		Py		Py		NAc (Outside)
Facial expression recognition		NBAc		NBAc		Py		Py		Py		Py		NAc (Outside)
Galvanic skin response		NBAc		NBAc		Py		Py		Py		Py		NAc (Outside)
Voice pitch analysis		NBAc		-		Py		-		-		-		-
Implicit association test		-		NBAc		-		-		-		Py		-
Self-reports		-		-		-		SR		-		-		-
Reaction time		-		-		-		BM		-		-		-

*BWA, Brain Wave Analysis; BIA, Brain Imaging Analysis; NBAc, No Brain Activity; NPy, Neurophysiological; Py, Physiological; NIm, Neuroimaging; SR, Self-reports; BM, Behavioral Measurements; NAc, Neuronal Activity; MNAc, Manipulate Neuronal Activity*.

There is a difference in the number and type of tools that authors include in each classification (see Table 1). All authors included tools such as EEG, fMRI, MEG, PET, fERS, ET, ECG, and GSR as consumer neuroscience tools (Wang and Minor, 2008; Bercea, 2013; Ramsøy, 2015; Boz et al., 2017; Harris et al., 2018; Lim, 2018). However, other tools such as fNIRS, TMS, SST, and VPA have not been mentioned in all classifications. Self-reports and reaction time are only discussed by Ramsøy (2015). This observation suggests that some authors identify as consumer neuroscience tools only those most commonly employed in neuroscience studies.

### 3.2. Popularity of Tools

Even though a large number of tools are proposed for consumer neuroscience research, it might be possible that the number of tools that are practically used for research is much lower. In our analysis, we did not find evidence of practical use for some of the proposed tools. In total, we analyzed 137 empirical research papers in consumer neuroscience research and we find that only seven tools are used in these studies. In particular, we see that some studies use tools that measure brain activity such as EEG (both traditional and wearable), fMRI and fNIRS. In addition, other studies use tools that measure physiological responses such as ECG, ET, GSR, and fERS. Surprisingly, tools such as MEG, SST, SPECT, PET, TMS, and VPA are not used in the studies in our search. However, it is noteworthy that many of these studies also use self-reports, questionnaires and/or reaction times to measure behavioral measurements. In addition, we found that approximately 18% of the total number of studies (25 studies) use a combination of two or more tools simultaneously.

Another important aspect to consider is the popularity of these tools in consumer neuroscience research. The literature suggests that several tools are in demand in the field. Some studies claim that the majority of consumer neuroscience research has predominantly used fMRI to measure brain activation in response to marketing stimuli (Smidts et al., 2014; Ramsøy, 2015; Harris et al., 2018). Other studies maintain that the most widely applied consumer neuroscience tool is eye-tracking followed by galvanic skin response (16%), facial recognition (11%), heart rate variability (9%), electroencephalography (6%), and electromyography (EMG) (4%) (dos Santos et al., 2015; Boz et al., 2017). A review of 34 neuroscience studies from 2001 to 2012 finds that fewer than 25% of the studies applied EEG, while almost 70% applied fMRI (Solnais et al., 2013; Sung et al., 2019). Similarly, Lim (2018) outlines that neuroimaging is the most popular category of tools for consumer neuroscience research (33 articles; 42.3%), followed by non-neuroimaging tools (16 articles; 20.5%). In particular, (Lim, 2018) outlines that the most popular brain recording method is fMRI and EEG is the second most popular tool.

**Figure 3:**
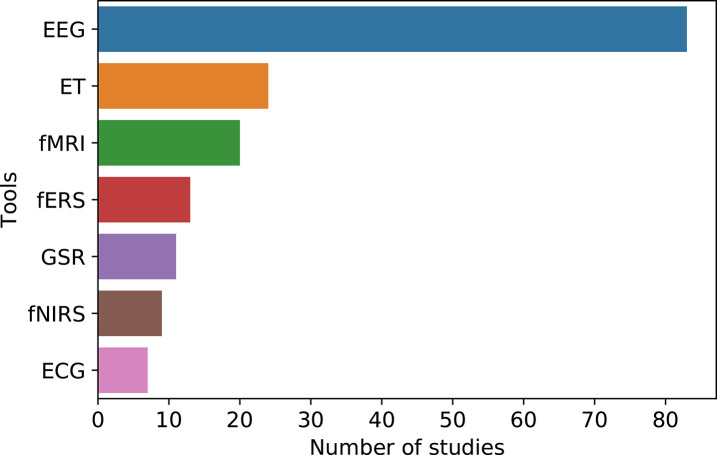
An overview of neuroscience tools used in *consumer neuroscience* studies.

In the literature review we found that most consumer neuroscience studies use EEG (both traditional and wearable). In total, EEG was used in 83 studies (approx. 60.5%), alone or in combination with other tools (see Figure 3). In 19 of these studies, researchers used wearable EEG. Eye tracking is the second-most used tool, appearing in 24 studies (approx. 17.5% of the studies). Interestingly, fMRI was used only in 20 studies (approx. 14.5%). As shown in Figure 3, fERS was found in 13 studies (almost 9.5%), followed by GSR (8%), fNIRS (almost 6.6%), and ECG (approx. 5%). These results are in line with a survey conducted by the Neuromarketing Science and Business Association (NMSBA) in 2018. The survey provides a ranking of the most to the least offered tools offered by neuromarketing vendors, namely EEG, ET, fERS and GSR. fMRI shows a small decline during the period 2014–2018 (Cherubino et al., 2019).

Overall, we identify seven tools that are used to measure consumer brain activity or physiological responses to marketing stimuli. Additionally, we find that many consumer neuroscience studies also employ traditional marketing tools such as surveys, questionnaires and self-reports. It might be possible that some tools are listed as consumer neuroscience tools but they have never been used before (e.g., MEG, PET, TMS, SST, SPET, VPA). We also believe that some of these tools such as PET and TMS should not be used in consumer neuroscience research. Whilst these tools are very helpful for medical diagnosis and treatment of mental diseases, they may be too invasive or have adverse effects (e.g., pain, fainting, seizures) that could be too high to be used in consumer neuroscience studies, thus exposing participants to unnecessary risks (Rossi et al., 2009; Dobek et al., 2015). Similarly, we believe that SPET and SST are not useful for consumer neuroscience research. SPET is very similar to PET and has the same disadvantages such as costs and high risk for the subject. In fact, SPET requires an injection of radiopharmaceuticals which is risky and arguably unethical. Finally, SST could not be considered as a neuroscience tool because it is a particular application of EEG. The available literature seems to support the view that fMRI and eye-tracking are the most used tools in consumer neuroscience studies. On the contrary, we find that EEG is the most popular tool used in consumer neuroscience studies by far, followed by eye-tracking. fMRI is only the third-most used tool in the field. There could be several reasons why fMRI is considered the most popular tool. Many papers were published 4–5 years ago, and might not reflect the current situation. Perhaps the fact that fMRI is broadly discussed in popular studies such as (McClure et al., 2004; Plassmann and Karmarkar, 2015; Venkatraman et al., 2015) gives the false impression that this tool is widely used in consumer neuroscience research.

### 3.3. Proposed Classification of Tools

**Figure 4:**
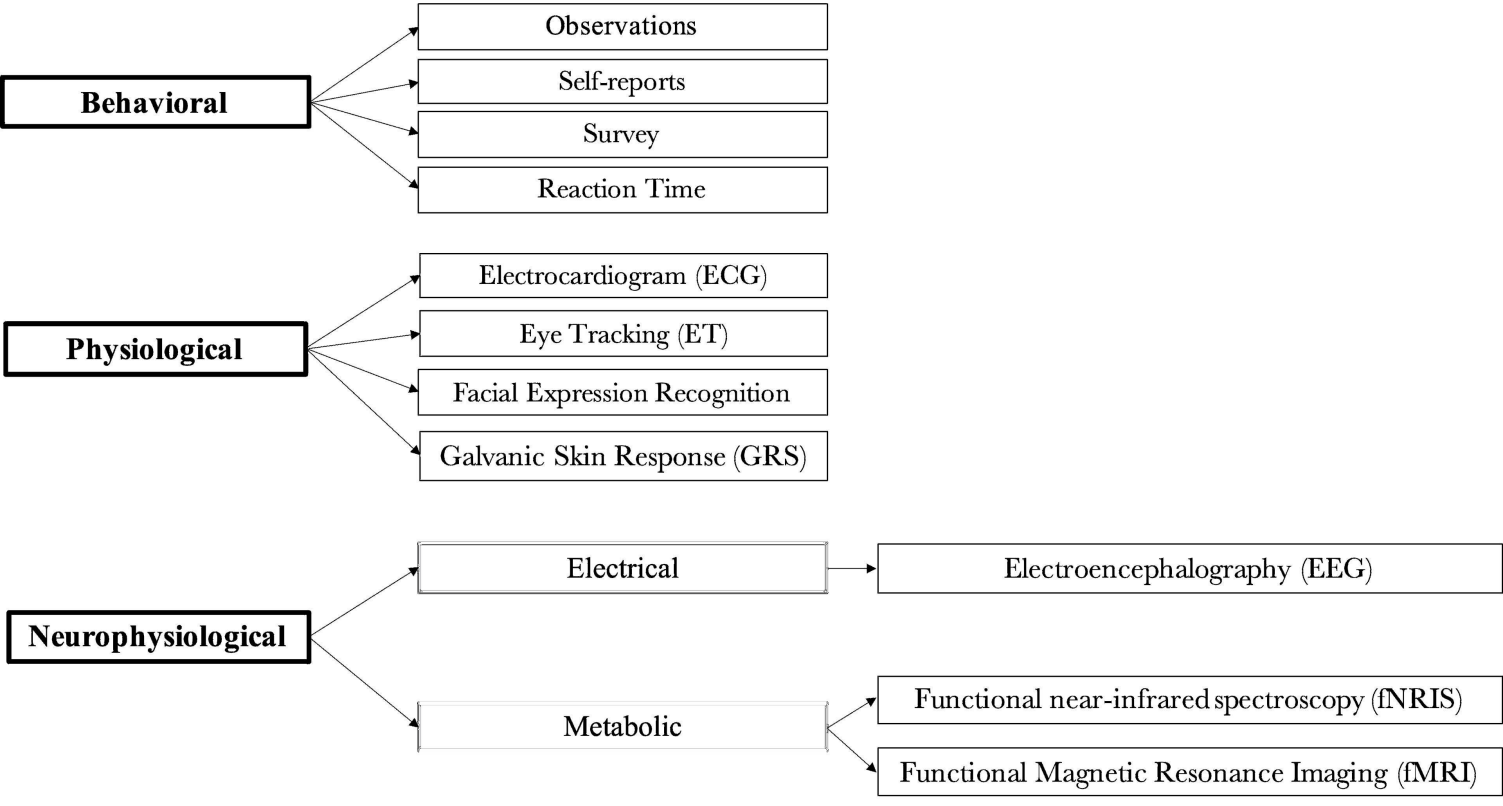
Proposed classification of consumer neuroscience tools.

Based on the above considerations, we propose a new classification following Isabella et al. (2015) and Ramsøy (2015) classifications. Figure 4 shows that consumer neuroscience tools can be divided in three categories, namely:

1. Behavioral,
2. Physiological,
3. Neurophysiological.

Behavioral tools (e.g., survey, observations, and RT) are part of our classification as they provide important information on behavior of consumers. These tools are also presently used in consumer neuroscience studies. We also do not differentiate between self-report and survey, as we believe self-report has a different purpose in this field than in psychology. In consumer neuroscience, self-reports are not used to assess personality but purchasing behavior and attitude of consumers (Paulhus and Vazire, 2007).

For both physiological and neurophysiological tools, we only include tools that are currently used in consumer neuroscience research. In our classification, physiological tools such as ECG, ET, fERS, and GSR can measure the autonomous functions of the body for which there is no direct or conscious control, such as blood circulation, blood pressure, heart rate and sweating (Kenning and Linzmajer, 2011). These tools allow researchers to measure the autonomous functions of the body (e.g., voluntary and involuntary body reflexes). Neurophysiological tools can directly measure consumers' brain activity and can be further divided into two categories: those that measure electrical activity (EEG), and those that measure metabolic brain activity (fMRI and fNRIS). The characteristics of physiological and neurophysiological tools are discussed in the next section.

## 4. Characteristics of consumer neuroscience tools

In this section, we discuss the details of the seven tools currently used in consumer neuroscience research (identified in section 3). We illustrate the cognitive processes which these tools can be used to investigate, and the main advantages and disadvantages for each tool. Secondly, we provide an estimate of average costs of procuring the tools and the average time it takes to perform an experiment. We also discuss the ease with which a tool can be used in tandem with another tool; we represent this as the *integration level* of a tool. Table 2 provides an aggregate view of all the above-mentioned characteristics for each of the neuroscience tools.

**Table 2:** Characteristics of Consumer neuroscience tools.

**Tool**	**Measurement**	**Advantages**	**Disadvantages**	**Equipment price**	**Time**	**Integration level**
EEG	Brain activity	Temporal resolution	Spatial resolution	€35K	1 hr	Medium
Wearable EEG	Brain Activity	Temporal resolution	Spatial resolution	€500 to €25K	30 min.	Medium
		Portable				
		Low cost				
fMRI	Brain Activity	Spatial resolution^a^	Temporal resolution^b^	€1M	1 hr.	Low
			Expensive			
			Non-portable			
			Ethical concerns			
fNIRS	Brain Activity	Low sensitivity to motion artifacts	Spatial resolution	€50K	1 h.	Medium
		Portable	Temporal resolution			
ET	Visual attention	Portable	Low flexibility	€100 to €30K	15 min.	High
	Pupil dilatation	Low cost	Glasses/Contact lenses			
	Fixations^c^					
ECG	Heartbeat	Portable	Slow signal	€10K	15 min.	Medium
	Blood flow	Low cost	Sensitivity to motion			
			Low individual usefulness			
fERS	Facial expressions	High flexibility	Low individual usefulness	€10K	15 min.	High
		Portable				
GSR	Skin moisture level	Portable	Low individual usefulness	€100 to €2K	15 min.	High

a *Spatial resolution refers to how accurately the measured activity is localized within the brain*.

b *Temporal resolution refers to how closely the measured activity corresponds to the timing of the actual neuronal activity*.

c *Amount and duration of fixations can be a metric for attention*.

### 4.1. Electroencephalogram

EEG can be considered the oldest neurophysiological tools, dating back almost a century (1924) (Murray and Antonakis, 2019). EEG is a non-invasive brain imaging method that detects brain electrical activity using different electrodes placed on the scalp (Berger, 1929). The electrodes measure small electrical potentials that reflect the activity of neurons within the brain. These potentials, whose amplitude is tiny, are then amplified, digitized and then transmitted to a personal computer for processing and storage. EEG measures the potential difference (i.e., the voltage) between two electrodes (Kane et al., 2017).

Electrodes can be made of various materials. Usually, EEG electrodes are made of metal plates and they are applied to the scalp using a conducting electrode gel. However, there has been an increase in the use of dry electrodes (no gel needed) in recent years (see next subsection). The positioning of the electrodes on the scalp has been standardized worldwide in the so called 10–20 international system (Klem et al., 1999).

Electrophysiological techniques usually have an excellent temporal resolution but poor spatial resolution (Burle et al., 2015). In general, EEG allows the detecting of the activity generated in the cortex only, and not in deeper brain structures because of the presence of the scalp's bones, the cerebrospinal fluid and the dura mater which act as filters for high frequencies. However, EEG spatial resolution (about 1 cm) can be increased using high-density EEG caps (128 electrodes or more).

In the temporal domain, EEG can measure Event Related Potentials (ERPs). ERPs are very small voltages generated in the brain in response to sensory, motor or cognitive events or stimuli (Blackwood and Muir, 1990). In the frequency domain, an EEG signal can be analyzed in different frequency bands, namely delta, theta, alpha, beta and gamma (Rahman et al., 2015). This analysis can be used in consumer neuroscience research to investigate cognitive processes such as attention, arousal, emotion, engagement, excitement, memory, reward, sensory perception and valence (see section 5) (Vecchiato and Babiloni, 2011; Ohme and Matukin, 2012; Di Flumeri et al., 2016; Rakshit and Lahiri, 2016; Dulabh et al., 2018).

EEG carries a relatively low cost for the equipment and tests. Depending on how many electrodes are used (and the technology employed), the estimated average cost is between ten and thirty five thousands euros per study (Lystad and Pollard, 2009). However, EEG also requires human resources for acquisition and analysis, as well as consumables. For setting up a consumer neuroscience experiment, an EEG technician and/or a data analyst to analyse data are required. A neurologist might also be required to check the quality and the reliability of the EEG signals. The average time for the preparation of a single experiment is between 30 min and 1 h, depending on the caps and the number of electrodes. The the time to set-up the experiment and the time to execute the experiment need to be added. EEG is characterized by a medium level of integration with the other neuroscience tools because it can be used in combination with various other tools, depending on the specific EEG hardware. Compared to other neurophysiological tools, EEG is more tolerant toward small physical movements made by the participant during an experiment.

### 4.2. Wearable Electroencephalogram

Electrophysiological techniques also include wearable EEG devices. A wearable EEG device consists of a portable cap, a base station and a pre-amplifier. The signal collected from electrodes is pre-amplified and transmitted wirelessly to the EEG amplifier through the base station.

Wearable EEG allows researchers to measure the same cognitive phenomena detectable with classical EEG. Wearable EEG is very popular in consumer neuroscience research due to increased subject mobility. We find that wearable EEG was used to investigate consumer behavior for e-commerce products and to measure users' emotional responses while watching products prior to purchase or product placement effectiveness (Murugappan et al., 2014; Yadava et al., 2017; Guo et al., 2018).

There are many different types of wearable EEG devices available on the market. Compared to traditional EEG, wearable EEG systems or headset devices are usually less expensive, because they have a low number of EEG channels. EEG headsets like Emotiv, OpenBCI, NeuroSky range from €500 up to a maximum of a few thousand euros (Lystad and Pollard, 2009). However, there are other more expensive wearable EEG devices (e.g., Nautilus from g.tec medical engineering) that have a higher number of electrodes and a higher performance. These devices can cost up to tens of thousands euros. Wearable EEG systems measure similar brain phenomena as the traditional EEG system. Compared to classical EEG systems, wearable EEG devices allow more liberty of movement for the subject. In addition, the average time for the preparation of the subject is less compared to traditional EEG (setup time of less than 5–6 min for Emotiv, Muse, and openBCI) (Qiu et al., 2019). Wearable EEG devices also have a higher level of integration compared to classical EEG devices because they are portable. Recent studies suggest that wearable EEG systems are capable of collecting neural signals, the quality of which is comparable with those collected by the traditional EEG systems in a controlled laboratory environment (Schiff et al., 2016; Kam et al., 2019; Qiu et al., 2019). However, studies show that wearable EEG systems are more prone to artifacts from muscle movements (Badcock et al., 2013; Ratti et al., 2017; Qiu et al., 2019). Some studies also suggest that the signal of wearable EEG might be delayed in comparison to traditional EEG systems (Qiu et al., 2019). For academic purposes, the use of more complex (thus, more expensive) EEG systems, together with expert personnel, is highly recommended, since it provides a higher quality and a more reliable signal.

### 4.3. Functional Magnetic Resonance Imaging

Functional Magnetic Resonance Imaging (fMRI) is a metabolic brain imaging method used to analyse regional, time-varying changes in brain metabolism (Ogawa et al., 1990; Bandettini et al., 1992; Kwong et al., 1992). fMRI measures the BOLD (Blood Oxygenation Level Dependent) response by tracking the changes in the blood flow indicated by the relative amounts of different forms of haemoglobin. fMRI was introduced by Ogawa in 1990, so it is can be considered a “younger” tool compared to EEG (Murray and Antonakis, 2019).

fMRI can be used to produce activation maps showing which parts of the brain are involved in a certain process. These metabolic changes can (1) be induced by the execution of a specific motor or cognitive task or as a result of a stimulus (task-related fMRI), or (2) be the result of uncontrolled processes in the brain at rest, in absence of a stimulus or a task (resting-state fMRI) (Ogawa et al., 1990; Bandettini et al., 1992; Kwong et al., 1992). The fMRI modality used in consumer neuroscience research is the task-related fMRI. Thus, activation maps are produced by comparing BOLD level contrast between active periods (e.g., performing a task or exposed to a stimulus) and rest periods.

fMRI is widely used due to its widespread availability, its non-invasive nature (does not require injection of a radioisotope or other pharmacologic agent), and good spatial resolution (about 1 mm). In contrast, it has a poor temporal resolution (Burle et al., 2015). fMRI allows for analysis of hemodynamic activity in small structures and even those brain structures that are deep in the brain, which are usually involved in emotional responses (e.g., amygdala and accumbens). fMRI is very expensive. In a hospital or research center setting, the typical cost of a fMRI scan is between €500 and 800, while a fMRI scanner costs not less than €1 million (Lystad and Pollard, 2009). To have valuable and reliable data, an fMRI experiment requires a psychologist or neuroscientist who sets up the fMRI task, an MRI technician who performs the experiment, a physicist who set ups the hardware to acquire the right brain responses and a biomedical engineer (or the same physicist) who analyses the data. The average time for the execution of an fMRI scan is about 30 min, which includes the time for the preparation of the subject and the time to acquire the images.

In the literature, we find that fMRI is the second most used neuroimaging tool in consumer neuroscience experiments. In fact, fMRI scanners are easily available compared to other neuroimaging tools. Several fMRI studies investigate many cognitive phenomena, such as sensory perception, attention, arousal, emotion, engagement, memory, reward and valence (McClure et al., 2004; Deppe et al., 2005; Esmaeili et al., 2011; Santos et al., 2012; Ruanguttamanun, 2014; Sebastian, 2014; Koestner et al., 2016; Shen and Morris, 2016). fMRI is a suitable tool to study consumer preferences for visual stimuli (e.g., videos, images). However, fMRI might not be suitable if researchers want to replicate exact “real-world circumstances,” for example touching the product or drinking from a glass. In fact, fMRI restricts participants' movements considerably, as they lie in a narrow tube (Alvino et al., 2019). Overall, fMRI may have a restricted external validity compared to other tools (e.g., EEG). There are some safety concerns on the use of this tool. Although fMRI can not be not considered an invasive tool, many subjects can suffer due to the noise of the machine (especially for fMRI sequences), the small space (claustrophobia, vertigo, nausea) and potential movement or heating of ferromagnetic objects in the body (e.g., pacemakers, surgical clip). For these reasons, subjects might not be able to complete the scan (Lystad and Pollard, 2009). Finally, fMRI has a low level of integration with other tools, due to the presence of magnetic fields. Thus, it can be integrated only with devices compatible with magnetic fields.

### 4.4. Functional Near-Infrared Spectroscopy

Functional near-infrared spectroscopy (fNIRS) is a non-invasive, metabolism-based brain imaging technique. It measures change in the blood's color when oxygen is delivered to brain tissue (only the top few mm of cortex) (Villringer et al., 1993; Boas et al., 2004; Burle et al., 2015). fNIRS, similarly to fMRI, is a BOLD-response technology that measures the changes in the relative levels of oxyhemoglobine (oxy-Hb) and deoxyhemoglobine (deoxy-Hb) during brain activity. When a subject performs a task, brain activity increases in those brain areas relevant to the task due to changes in oxy- and deoxy-Hb (Ernst et al., 2013).

fNIRS is relatively easy to use as its technology supports fast data acquisition from numerous positions (Ehlis et al., 2005; Sitaram et al., 2009). Its temporal resolution is relatively good (few seconds), but it seems to be lower in comparison to EEG. In addition, fNIRS cannot be applied when the focus of the research is the investigation of cognitive processes which rely on deeper structures of the brain. In fact, fNRIS's spatial resolution is very low, making it difficult to distinguish between cortical areas that are positioned close to each other.

We find that the use of fNIRS in consumer neuroscience research is very recent. fNIRS is used to investigate consumer attention, arousal, emotions, sensory perception, and valence, especially for mobile technologies (Plichta et al., 2011; Ernst et al., 2013; Cakir et al., 2018; Krampe et al., 2018). In general, fNIRS is easily available for economic researchers (Shimokawa et al., 2009; Weiskopf, 2012; Kober et al., 2014). fNIRS is a noise-free neuroimaging tool, and thus it can be very useful in studies in which auditory stimuli play an important role (unlike fMRI) (Plichta et al., 2011). The low sensitivity of motion artifacts also creates strong potential for fNIRS and its application in real world scenarios (Nambu et al., 2009; Lloyd-Fox et al., 2010; Kober et al., 2014).

fNIRS equipment is portable and inexpensive compared to other blood-flow measurement technologies, such as fMRI. fNRIS's price is around €10.000, but it can go up to €200.000 (depending on the technique and associated electronics). An fNIRS experiment needs at least an expert technician to perform the experiment and a data analyst. The average time needed to perform an fNIRS session is about 1 h, which includes the time for the preparation of the subject (measurements of anatomical landmarks, positioning of the cap, digitization of the positions of the sensors) and the time to perform the experiment, which usually takes place in a block-design paradigm experiment, alternating active periods with rest periods. fNIRS, particularly the portable version, is characterized by a good level of integration with other consumer neuroscience tools.

### 4.5. Eye Tracking

An eye tracker (ET) is a device that allows the measurements of eye positions, eye movement and pupil dilatation. Researchers can use ET to measure how information on the screen is related to behavioral and emotional responses (Wang, 2011). ET measurements are fixation (looking at a specific place), length of fixation (how long a person looks), saccades (fast eye movement) and pupil dilation responses (changes in pupil size) (Wang, 2011). ET technologies usually measure eye gaze and movements at specific (and fixed) points on images or videos. However, there are more advanced devices that allow the automatic tracking of the user's head position and movement (Zurawicki, 2010). Such enhancements make the measurement process more subtle, with very little or no interaction between the researchers and their subjects. In the market, there are two types of ET devices available, namely fixed ones, which are integrated in another device (i.e., a computer monitor) and wearable ones, which consist of spectacles with integrated cameras.

ET has been widely used in consumer neuroscience research to study visual behavior (e.g., fixation, gaze, pupil dilatation), customers' visual attention mechanisms and consumers' engagement (Zamani et al., 2016; Ungureanu et al., 2017). ET has several advantages: it is portable, non-invasive, simple to use and relatively inexpensive. ET has a cost ranging between €100 and 30,000 euros, depending on the level of the technology and whether the software to acquire and analyse data is included^1^. An ET experiment needs only a technician and, eventually, a data analyst. The average time needed to perform an ET experiment is about 15 min since the subject set-up is very fast. This time covers only the time needed to perform the experiment by the subject. Not all the ET has a high flexibility as some ET models might not work efficiently with glasses and contact lenses. ET is also characterized by a high level of integration with other tools due to its portability and because it is a “ready-to-use” device. To have more reliable results, ET should be used in combination with other tools.

### 4.6. Facial Expression Recognition Software

Facial expressions are important metrics of subjects' emotions. They are usually divided into two main categories: observable and unobservable (Fortunato et al., 2014). The analysis of facial expression is divided into two main classes, namely facial electromyography (fEMG) and facial expression recognition software. The former category measures voluntary and involuntary facial muscle movements reflecting emotional reactions toward a marketing stimulus (Bercea, 2013; Cherubino et al., 2019). The latter category is based on the use of specific software to record and analyse facial expressions based on decision classifiers, and is able to predict a purchase substantially above the chance level.

Facial expression recognition software (fERS) can be used to measure positive or negative reactions to marketing stimuli (e.g., videos). fERS is a valuable tool to investigate consumers' excitement, engagement, emotions and valence (Ângelo et al., 2013; Hamelin et al., 2017; Hernández-Fernández et al., 2019).

There are many facial recognition software platforms in the market. For example, Software iMotions Neuromarketing combines different sensors and is able to detect a subject's attention, valence and emotions (iMotions, 2020). In contrast, the Facial Action Coding System is an online platform to measure emotions and understand human behavior through face analysis^2^.

The costs of an fERS are highly variable and depend mainly on how many integrated sensors the platform is able to manage. If we consider only the platform (excluding the sensors), the cost of such software can be around a few thousand euros. Conducting an experiment using facial recognition expression requires one technician to acquire face data and a technical expert for data analysis. The average time needed to perform such an experiment is 15–20 min. No subject preparation is required. Finally, this tool is characterized by a high level of integration with other consumer neuroscience tools. fERS is portable, very easy to use and can manage the data of various sensors. Using only fERS has a limited application for consumer neuroscience research, as it does not measure important cognitive processes such as attention, memory and sensory perception.

### 4.7. Electrocardiogram

Electrocardiogram, also called ECG or EKG, is a tool used to measure the electrical activity of the heart (Stern et al., 2001). Heart rate variability (HRV) is the physiological phenomenon of variation in the time interval between heartbeats and it reflects the activation of the sympathetic and parasympathetic parts of the autonomic nervous system. Several studies reported that changes in the hearth rate (HR) might be correlated with changes in the emotional state of a subject (arousal and valence) (Ha-Brookshire and Bhaduri, 2014; Valenza et al., 2014; Baraybar-Fernández et al., 2017).

ECG is a very simple, portable, non-invasive, widely used and accessible tool that has a good temporal resolution. To use ECG, researchers need only the device, a monitor and a computer to synchronize the ECG signal (electrophysiological response) with the stimulus. ECG has quite low costs (around tens of thousands euros). The average time needed to perform an experiment using ECG is about 15 min, because subject preparation is very fast. It does require specialized personnel to acquire and analyse the signal. However, ECG has limited applications because it should be used in combination with other consumer neuroscience tools.

### 4.8. Galvanic Skin Response

Galvanic Skin Response (GSR) is a physiological tool that measures the electrical conductance of the skin through one or two sensor(s), usually attached to some part of the hand or foot (Nourbakhsh et al., 2012). The physiological basis of the galvanic skin response is a change in autonomic tone (skin and subcutaneous tissue) in accordance with the emotional state of the subject. It is used to measure skin resistance, conductance, and stress level in the body.

GSR is also considered a sensitive tool for measuring changes in subject's arousal and valence (Ravaja, 2004; Ayata et al., 2016). Skin conductivity (SC) can reveal an activation in the autonomic nervous system (ANS) (Nourbakhsh et al., 2012). Because an increase in the activation of the ANS is an indicator of arousal and valence, SC can be used as a measure of arousal and valence (Vecchiato et al., 2014; Ayata et al., 2016).

GSR equipment is easy to set up and transport, making it ideal for work in the field, for example, for assessing levels of emotional arousal during in-store shopping experiences. However, GSR has a low temporal resolution. In fact, different skin types can create variability in responses across subjects, thus making results difficult to aggregate. In addition, GSR is very sensitive to artifacts. In fact, test subjects can freely move their hands and body which may result in artifacts or false readings in the GSR. During data analysis, artifacts can be detected and removed by applying post-processing tools. The major limitation of GSR is that it can only measure changes in subject's arousal and valence but it cannot determine the direction or the valence of an emotional reaction. GSR requires at least a computer to correlate the stimulus with GSR software to have reliable results.

The average cost of a GSR amplifier is in the order of a few thousand euros. A GSR experiment requires one technician to acquire GSR data and a technical expert for data analysis. The average time needed to perform a GSR experiment is about 15 min because subject preparation is very fast. Finally, GSR is characterized by a high level of integration with other consumer neuroscience tools as it is portable and very easy to use (Ozkul et al., 2019).

## 5. Main Applications in Marketing

Consumer neuroscience has the potential to support marketing research on how cognitive processes (e.g., perception, memory, attention) originate in the brain and identify the brain areas involved in the explication of cognitive functions underlying marketing-relevant behavior. This section focusses on how consumer neuroscience tools are used to study consumer behavior. On the basis of the literature survey we identify the following marketing-related topics where these tools are used: advertising, branding, product experience, online experience, product development and product pricing.

### 5.1. Advertising

We find that almost 45% of the studies (61 studies) investigate the impact of advertising on consumer behavior (e.g., preferences, satisfaction), emotions (e.g., positive, negative), and cognitive processes (e.g., attention, memory, engagement). Consumer neuroscience research analyses how consumers experience, process and assess advertisements (Astolfi et al., 2008; Ohme et al., 2010; Treleaven-Hassard et al., 2010; Morillo et al., 2015; Soria Morillo et al., 2016; Cha et al., 2019; Golnar-Nik et al., 2019; Kaklauskas et al., 2019; Kumar et al., 2019; Mahamad et al., 2019; Shaari et al., 2019). Studies focus on all types of advertising, i.e., brochure, billboard advertising, endorsements by spokespersons, movie trailers, television advertisement, social advertisement, Youtube videos and websites (Ohme and Matukin, 2012; Vecchiato et al., 2012a,b; Randolph and Pierquet, 2015; Hamelin et al., 2017; Jin et al., 2017; Missaglia et al., 2017; Kong et al., 2019).

Depending on the tool used, we can get information on how consumers process advertising stimuli. For instance, EEG can be used to evaluate the effectiveness of advertising by measuring the brain activity within milliseconds (Vecchiato et al., 2011; Kong et al., 2013; Pileliene and Grigaliunaite, 2017b). In particular, EEG oscillations, measured through the changes in spectral power of EEG for some certain frequency bands, and in specific brain regions, while investigating in the frequency domain, or, while investigating in the time domain by looking at the derivation of particular event-related potential (ERPs), can indicate a higher or lower level of attention/memory/engagement of participants for a different type of advertisements (e.g., movie trailers, anti-binge-drinking campaigns, Boksem and Smidts, 2015; Gountas et al., 2019, see Table 3).

**Table 3:** Neurophysiological tools: applications in marketing, products, and services.

**Tool**	**Cognitive processes**	**Behavioral measurements**	**Marketing application**	**Product or service**
	Attention	Age difference	Advertisement	App
EEG	Arousal	Consumer preferences	Brand	Brand names
	Emotion	Consumer satisfaction	In-store experience	Car
	Engagement	Gender difference	Online experience	Clothes
	Excitement	Intention to purchase	Price	Coupon
	Memory	Sentiment score	Product characteristics	Drink
	Reward	Purchase behavior	Product experience	Food
	Sensory perception		Product quality	House
	Valence		Promotion	Mobile phone
				Movie trailers
				Music
				Shoes
				Perfume
				Text-to-speech
				Television commercial
				Video
				Wine
	Attention	Age difference	Advertisement	Accessories and Bags
	Arousal	Consumer preferences	Brand	Car
	Emotion	Consumer satisfaction	In-store environment	Celebrity
	Engagement	Gender differences	Online experience	Clothes
	Excitement	Sentiment score	Price	Coffee mug
Wearable EEG	Memory	Purchase behavior	Product characteristics	Food
	Sensory perception		Product experience	Shoes
	Valence		Promotion	Sport activities
				Spoken-person
				Television commercial
				Video
	Attention	Consumer preferences	Advertisement	Book
	Arousal	Consumer satisfaction	Brand	Brand logo
	Emotion	Gender difference	Price	Car
fMRI	Engagement	Sentiment score	Product characteristics	Food
	Memory	Purchase behavior	Product experience	Wine
	Sensory perception		Product quality	
	Reward		Promotion	
	Valence			
	Attention	Consumer preferences	Advertisement	Drink
	Arousal	Purchase behavior	Brand	Lipstick
fNIRS	Emotion		Price	Paper and display media
	Sensory perception		Product characteristics	Music player
	Valence		Product experience	Music
			Product quality	

Another important tool used to investigate consumers' responses to advertisement is eye tracking. ET allows researchers to determine consumers' visual attention though heat maps, scan path and eye fixations (see Table 4). Measuring where and for how long a person is looking at a specific advertisement could provide important information on the level of attractiveness (e.g., high number of fixations vs. low number of fixations) and visibility (e.g., right size of product in the advertisement) of an advertisement (e.g., Youtube video, product image) (Venkatraman et al., 2015; Zamani et al., 2016).

**Table 4:** Physiological tools: applications in marketing, products and services.

**Tool**	**Cognitive processes**	**Behavioral measurements**	**Marketing application**	**Product or service**
	Attention	Age difference	Advertisement	Brand logo
	Excitement	Consumer preferences	Brand	Car
	Engagement	Gender difference	In-store environment	Clothes
			Destination marketing	Drink
Eye Tracker			Online experience	Food
			Product characteristics	Household products
				Spoken-person
				Video
				Website
				Wine
	Arousal	Age difference	Advertisement	Brand logo
	Emotion	Consumer preferences	Brand	Car
	Valence	Gender difference	In-store environment	Clothes
Galvanic Skin Response			Online experience	Food
			Product characteristics	Store color and light
			Product experience	Perfume
				Television commercial
				Video
	Arousal	Age difference	Advertisement	Advertisement spot
Electrocardiogram	Emotion	Consumer preferences	Brand	Perfume
	Valence	Gender difference	Online experience	Television commercial
				Video
	Emotion	Age difference	Advertisement	Brand logo
	Engagement	Consumer preferences	Brand	Food
	Excitement	Gender difference	In-store environment	Google glass
Facial Expression Recognition Software	Valence	Sentiment score	Online experience	Music
			Price	Store color and light
			Product characteristics	Spoken-person
			Product experience	Television commercial
			Product quality	Video

fMRI is used to test advertisement effectiveness for product images. In particular, fMRI can be used to investigate the hemodynamics response (brain activations) in brain regions responsible of these phenomena (Morris et al., 2009; Kühn et al., 2016; Shen and Morris, 2016; Jung et al., 2018; Casado-Aranda et al., 2019). As it has a low temporal resolution, fMRI might not always be suitable for measuring brain activity during the view of audio-visual stimuli, due to the quick changes in the scenes (Zurawicki, 2010).

Finally, tools such as GSR, ECG and facial expression recognition software can be used to measure emotional responses (e.g., sadness, joy, fear) to advertisement (Guixeres et al., 2017; Missaglia et al., 2017; Goyal and Singh, 2018). For instance, these tools can be used to test how the effects of high emotional and low emotional advertising in social advertisement (e.g., safe driving video) or YouTube videos (e.g., professional bank speakers) can affect consumer behavior (e.g., safe driving attitude) and preferences (e.g., video's popularity) (Lewinski, 2015; Hamelin et al., 2017).

Overall, we find that there are three different areas that could benefit from the application of consumer neuroscience tools in advertising. In particular, we can use these tools to test:

- Advertising effectiveness
- Target audience
- Salient features.

Advertising effectiveness measures how well a company's advertising (or advertising campaign) reaches and generates interest among customers or potential customers. Advertising effectiveness is usually measured in relations to sales (Wells, 2014). However, consumer neuroscience research can provide researchers and companies with measurements that give a better understanding of how consumers respond to an advertisement (e.g., positive or negative). It helps to study how customers acquire and process advertisement information and how behavioral measurements can be linked to cognitive and neuronal processes. In particular, consumer neuroscience tools can help to study those behavioral measurements (e.g., preference, satisfaction) and cognitive processes (e.g., attention, engagement and memory) that are useful to investigate when determining how an advertisement can catch consumers' attention, or is encoded in their memory. Many studies focus on post-design application. However, consumer neuroscience tools can also be very useful in pre-testing an advertisement—selecting spokespersons (e.g., female), the right color temperature and type of music to use in advertisement campaigns, for example (Wang et al., 2016; Pileliene and Grigaliunaite, 2017a; Avinash et al., 2018; Daugherty et al., 2018).

Another important aspect to consider in advertising is selecting a company's target audience; the group of customers or potential customers to whom a company addresses its product or service. Finding the right target audience is crucial to improve a campaign's efficiency. Consumer neuroscience tools can be used to test the impact of advertisements on different target audiences (e.g., male, female, young, adult, user, no user). For instance, Añaños (2015) used ET to measure differences in the level of attention in elderly adults, compared with young people during TV commercials. The results show that elderly people show a lower level of attention for integrated content compared to young people (Añaños, 2015). This finding suggests that advertising formats (low, medium, high level of disruption) can have a different impact on recognition between different target groups.

Finally, consumer neuroscience tools can be used to test an advertisement's salient features. An advertisement with good salient features is capable of holding a consumer's attention and it has great memorizing value. Consumer neuroscience tools measure how different versions of an advertisement or changes in a TV commercial or video (e.g., length, speed, complexity of the scene) can affect consumers' preferences, attention or recall for the advertisement (Zurawicki, 2010). A good example of salient features testing is the study of Ohme et al. (2009). The study measured the impact of two versions of a skin care product advertisement using EEG, EMG, and GSR. Results show that people were unable to identify any differences between the two advertisements at a conscious level (when asked). However, the neurophysiological measurements show that there was an increase in alpha bands (detected using EEG) and arousal level (detected using GSR) during the extra scene (model's gesture) in one version. EMG also revealed a significant difference in the facial muscle activity while watching the alternative scenes of the ad, specifically the additional scene provoked a higher level of corrugation muscle activity. This finding suggests that consumer neuroscience tools can add useful and important information to study an advertisement's salient features.

### 5.2. Branding

In today's economy, consumers buy emotional experiences rather than products and services (Hultén, 2011). The emotional linkage and experience associated with a brand is extremely important in building strong brands. Consumers' expectations on how much enjoyment they will derive from consuming a certain brand does not depend on the real value of the brand. Consumer expectations derived from consuming a brand are a mix of emotional and cognitive factors, and these expectations are usually formed on an unconscious level (Plassmann et al., 2012). Thus, consumers often find it difficult to describe why they enjoy a specific brand and/or why they are able to remember a brand. Consumer neuroscience tools can help companies to study emotional and neuronal processes while consumers choose, experience and remember brand's name or logos.

We identify two ways in which consumer neuroscience can contribute to a better understanding of the psychology of branding.

- Brand choice
- Brand loyalty.

People often choose products based on their perceived value, thus what the brand represents rather than the brand's actual value (Airey, 2009). Consumer neuroscience tools can be used to investigate the emotional component underling brand evaluation and choice, for example, differences between two similar brands (e.g., Pepsi vs. Coke) (Ma et al., 2007, 2008; Lucchiari and Pravettoni, 2012; Reimann et al., 2012a,b; Pop et al., 2013; Al-Kwifi, 2016; Guo et al., 2018). Bosshard et al. (2016) used EEG to test whether or not liked and disliked brands are further associated with different motivational aspects. The findings of this study suggest that liked brands elicited significantly more positive going waveforms (late positive potentials) than disliked brands over right parietal cortical areas. This result might infer that liking a brand influences consumers' electrical brain activity. In addition, consumer neuroscience tools can help to investigate how different brands are encoded, consolidated, and retrieved from the consumer's memory (McClure et al., 2004; Wang et al., 2012; Kačániová and Vargová, 2017; Levrini et al., 2019; Sung et al., 2019). For instance, remembered value refers to how different brand associations are encoded, consolidated, and retrieved from the consumer's memory (Plassmann et al., 2012). Using consumer neuroscience tools can help companies create and design strong brands that are easy to remember and have a strong emotional appeal.

Brand loyalty can be defined as the positive attitude toward a brand (Ferrell and Hartline, 2012). Customers that are loyal to a brand have a consistent preference for that brand, despite other situational and marketing factors that have the potential to induce switching behavior (Oliver, 1999). Consumer neuroscience tools can help researchers to study the neuronal and cognitive processes underlying brand loyalty (McClure et al., 2004; Schaefer and Rotte, 2007; Lin et al., 2010; Plassmann et al., 2012). For instance, Plassmann et al. (2007) investigated the differences in neural activation between loyal and disloyal customers of a store during purchasing decisions. Based on psychological theories, the authors assumed that for loyal customers the exposure to the store brand would modulate their decision via an emotion-based path. The authors found that customers who are loyal to a brand show more activation in the striatum compared to customers who are less loyal, even if they are buying identical clothes. The finding of this study also suggests that loyal customers usually establish an emotional bond with the physical brand stores, which might be the underlying psychological driver of their repurchases.

### 5.3. Online Experience and Website Optimization

Living in the digital era offers new opportunities for both consumers and companies. Consumers usually have a very wide offer of products and services worldwide, which is accessible 24/7. Similarly, companies have easier access to customers' information (e.g., location, age). Companies can also now reach their customers more easily by automating customer services (e.g., chatbot) and creating personalized messages for customers (e.g., personalized emails). Thus, the use of digital channels in business contributes to creating a very dynamic and competitive environment. Understanding the psychology behind online consumer behavior is key to compete in today's markets.

Consumer neuroscience tools can improve the online customer experience by testing the effectiveness of digital marketing campaigns for:

- Online rating and electronic word-of-mouth (eWOM) and
- Website and app optimization.

The increasing use of the Internet affects how people gather information. Nowadays, consumers can receive information on product and services more easily and faster compared to 20 years ago. In particular, online reviews, and eWOM (e.g., blogs) have become a significant and common way to acquire information about a company or a brand. Since online reviews and eWOM can influence other customers or potential customers, companies try to adopt strategies that can affect customers' rating behavior (Wang et al., 2018). Consumer neuroscience tools can be used to assess the effectiveness of online strategies by analyzing the emotional responses of consumers to online ratings and reviews (Wang et al., 2018; Hernández-Fernández et al., 2019). Measuring physiological and emotional responses associated to different strategies (e.g., a discount coupon for a five-star rating) may help to determine (1) how consumers perceive digital marketing strategies (e.g., aggressive, invasive) and (2) determine the emotional responses (positive, negative, surprise) to specific reviews or discussion topics (e.g., environment, service).

Website and App optimization is another important aspect of digital marketing strategies. Designing a good website or App is critical to improve the quality of the user experience (e.g., customization, online appointments, orders). An effective website or App should take into account different factors such as visual design and layout, usability, speed, content and search. Consumer neuroscience can be useful to test consumers' emotional responses to these factors (e.g., layout) and optimize websites and Apps (Chai et al., 2014; Jin et al., 2017; Yadava et al., 2017; Casado-Aranda et al., 2019). For instance, ET is an effective tool to test how users interact with a website (e.g., Mercedes-Benz) (Etzold et al., 2019). In fact, ET heat maps help identify shortcomings and hidden information (e.g., search button) when customers navigate on a website.

### 5.4. Pricing

One important element of the marketing mix that may influence consumer behavior is price. In the literature, only a few studies investigate how price influences cognitive and neural mechanisms. In particular, consumer neuroscience tools are used to study the effect of price on experienced quality by consumers (e.g., positive or negative expectations).

Consumer Neuroscience tools can be used to study three important aspects in pricing strategy:

- Price fairness
- Premium pricing
- Promotion.

Price fairness refers to the process that leads consumers to define if a price is reasonable or not, compared to one or more prices (Xia et al., 2010). Usually, people want to pay less for products, however, prices below a lower price-threshold may signal poor product quality. Instead, prices above an upper threshold may be considered as too high (price deception) (Fu et al., 2019). The biggest challenge for companies is to set a “fair” price for customers without altering their perception of quality (Cakir et al., 2018; Wang et al., 2018). Consumer neuroscience tools can be used to assess how consumers experience price and the effect of price based on their brain activity (e.g., positive, negative). For instance, consumer neuroscience tools can be used to study the difference in brain activity when consumers are exposed to low, optimal, and upper price-thresholds (Linzmajer et al., 2011).

Premium pricing, also known as prestige pricing, is used to influence consumers' expectations of the product and ultimately shape product experience (Almenberg and Dreber, 2011). Premium price is the marketing practice of selling a product for a higher price than competitors. Consumers usually associate the high price with higher quality. Thus, premium price creates an illusion of higher quality. Many marketing studies examine how knowledge about the price of a good affects how the good is experienced (Steenkamp et al., 2010; Almenberg and Dreber, 2011; Sellers Rubio et al., 2016). Consumer neuroscience studies also investigate how people enjoy consuming identical products (e.g., wines, underwear) more when they have a higher price. For instance, Plassmann et al. (2008) used fMRI to examine how tasting the same wine with different price (low and high) can effect consumers' preferences and brain activity. Their findings suggest that drinking wine with a higher price increase consumers' consumption enjoyment. In fact, the study found an increase in the medial orbitofrontal cortex (mOFC) due to changes in the price of a wine. mOFC is correlated with behavioral pleasantness for odors, tastes and music. The results suggest that increased activity in the mOFC leads to a change in the actual experienced pleasantness derived from its consumption (Plassmann et al., 2008).

Finally, consumer neuroscience tools can be used to assess the effect of promotion on buying behavior for coupons, promoted and non-promoted products, and unstructured or structured sales techniques (Jones et al., 2012; Muñoz-Leiva et al., 2019). Using EEG, Jones et al. (2012) measured consumers' brain activity during buying decisions for promoted (e.g., 15%) and non-promoted products. The author investigated how consumers (both male and female) with different characteristics (high and low math anxiety) respond to different buy/no buy decisions for promoted and non-promoted products. Using ERPs, Jones et al. (2012) tested if high math anxiety leads to a risk-reduction buying mentality (buy/no-buy decisions) compared to low math anxiety and if this difference was influenced by gender. The results showed a difference in the brain activity of High MA consumers (process price information relatively less fluently) compared to Low MA consumers (process price information more fluently) during buying decisions (e.g., larger LPC was detected for Low MA females under no promotions for non-buys compared to High MA females). This may be due to the adoption of a decision style which seeks to reject offer prices whereas High MA females may assess prices via attempting to confirm them (Jones et al., 2012). In addition, larger FN400 amplitudes associated with enhanced conceptual processing) were linked to purchases under promotions among High MA females and Low MA males (Jones et al., 2012). The reverse effect occurred for High MA females under no promotions (larger FN400 were4 found for non-buys than buys) (Jones et al., 2012). Overall, the results suggest that math anxiety, promotion format, and gender influence both consumer behavior and brain activity during consumer buying decisions.

### 5.5. Product Development

Consumer neuroscience research can help companies to design and develop desirable products by studying consumers' preferences for a product's internal and external characteristics. External characteristics (or aesthetics) refer to the visual, tactile and formal attributes of a product, such as color, shape and materials (Rindova and Petkova, 2007). The literature suggests that external characteristics can strongly influence consumers' perception and preference of products. For instance, colors can influence moods and feelings (positively or negatively), and consequently a consumer's attitude toward certain products (Singh, 2006). Internal characteristics refer to those specific and physical properties (e.g., ingredients, durability, taste) that cannot be changed without altering the nature of the product itself (Olson, 1976). However, consumers' perceptions of internal characteristics may be modulated by external characteristics. Consumer Neuroscience tools can be used to investigate how product characteristics influence consumers' sensory perceptions and preferences (Milosavljevic et al., 2012; Touchette and Lee, 2017; Muñoz-Leiva and Gómez-Carmona, 2019; Ploom et al., 2019). In particular, studies investigated how design, visual and tactile attributes influence consumers' brain activity. Consumer neuroscience tools also allow researchers to study how consumers assess product quality. Research on product characteristics mostly focussed on post-design applications. In the future, consumer neuroscience tools might be used to help companies developing new products in terms of:

- Product characteristics
- Product quality.

Consumer neuroscience research also focusses on the impact of external characteristics (e.g., packaging, color) on consumers' physiological and neuronal responses. For instance, (Reimann et al., 2010) used fMRI to examine the effect of different packaging designs (decorated vs. standard) on consumers' engagement and affective processing (reward). The results of that study show that intense emotional responses, such as the view of decorated packages, elicited strong affective processing. In fact, brain areas related to the reward system (nucleus accumbens and the ventromedial prefrontal cortex) were active during the view of decorated packaging. These results suggest that decorated packaging triggers reward mechanisms and choice compared to plain and simple packaging design.

Understanding how consumers assess product quality is important to study as it may influence consumer buying behaviors. Product characteristics can affect how consumers perceive product quality (Olson, 1976). The evaluation of product characteristics is a cognitive process that modulates attention, thus, consumer neuroscience tools can be used to analyse neural processes that occur during product evaluation. For instance, EEG can be used to test attentional mechanisms during consumers' evaluation of product quality for different products (e.g., shoes, music, wine) due to its high temporal resolution (Yilmaz et al., 2014; Gkaintatzis et al., 2019). Instead, fMRI can also be used to measure how consumer expectations of product quality are assessed based on product characteristics and individual preferences (Muñoz-Leiva and Gómez-Carmona, 2019).

### 5.6. Product Experience

Product experience occurs through interaction between the consumer and the product, physically or visually (Brakus et al., 2009). During the product experience, consumers are typically asked to reflect on the physical and emotional reactions induced by product experience. Another important contribution of consumer neuroscience research is the study of sensation or perception during product experience. Sensation is the activation of sensory receptor cells at the level of the stimulus (e.g., taste receptors). Perception is the processing of sensory stimuli into a meaningful pattern (BCcampus, 2018). The process of sensory perception begins when a product (e.g., perfume) stimulates our sense organs (e.g., tongue, nose, skin). Consumer neuroscience studies how people process, recognize and characterize sensory information through the five major senses, sight, smell, taste, touch and hearing. Several consumer neuroscience studies investigated how appearance, odor, flavor, taste and texture attributes can influence sensory perception and preferences of different products (e.g., food, lipsticks, music, water, wine) (Nittono and Watari, 2017; Tanida et al., 2017; Avinash et al., 2018; Hsu and Chen, 2019; Meyerding and Mehlhose, 2020). For instance, (Alvino et al., 2019) used EEG to measure brain activity and individual preferences for red wines during two tasting sessions (blind and normal). The findings of that study suggest that EEG is a useful tool to study brain activity during product experience due to its high temporal resolution and superior manoeuvrability compared to other consumer neuroscience tools.

Overall, consumer neuroscience tools can be used to study different types of product experience:

- In-store experience
- Destination marketing and Tourism.

In-store experiences occur when a consumer interacts with a store's physical environment (Kerin et al., 1992; Brakus et al., 2009). In-store experience does not necessarily lead to sales but it can encourage visitors to the store. Thus, improving in-store experience can help companies to be more competitive by offering experiences that are not available online. The effect of in-store experience can be tested by measuring how in-store variables, such as color, light, or music on consumers influence consumer buying behavior (Berčík et al., 2016; Ozkul et al., 2019). For instance, consumer neuroscience tools can be used to study the effect of light intensity and color temperature on purchasing decisions for fresh food (e.g., meat, dairy products) and beverages (wine, alcohol) (Horská and Berčík, 2014). Those studies used portable devices, like wearable EEG and ET, to measure consumers' physiological responses.

Finally, consumer neuroscience tools are used to test tourism satisfaction and measure physiological reactions during tourism experiences (Ma et al., 2014; Muñoz-Leiva et al., 2019). Consumers usually choose a travel destination not only based on the style of vacation they want but also considering the time, price and accommodation. However, emotional reactions to tourist destinations play a big role in their choices. Thus, travel agencies usually try to create campaigns that emotionally resonate with consumers, to create a deep emotional bond between the destination (e.g., city, hotel) and the customer. Consumer neuroscience tools can be used to assess which elements of the destination marketing campaign can trigger positive or negative emotions. For example, (Bastiaansen et al., 2018) using EEG to evaluate the effectiveness of tourist destination marketing content in movies on Bruges and Kyoto. We find that the number of studies on tourism experience is limited. However, consumer neuroscience tools might be used in the future to assess how consumers experience tourism and hospitality services, for instance, room and spa services.

## 6. New Methods in Consumer Neuroscience Research

We find two ready-to-use platforms that allow the use of several consumer neuroscience tools (e.g., ET, GSR) simultaneously. Several studies have already used a combination of different consumer neuroscience tools (e.g., ET, ECG). Using ready-to-use platforms may facilitate researchers to measure behavioral, physiological, and neurophysiological responses in a single experiment. Measuring brain activity and physiological responses simultaneously could help researchers to link cognitive and emotional aspects with neuronal processes during product experience (e.g. beverage tasting), online and offline purchase decisions, product quality assessment and brand exposure. Furthermore, ready-to-use platforms might also ensure a more reliable timing of signal acquisition, and direct synchronization between stimuli and responses (iMotions, 2012). Thus, these advantages could contribute to creating more realistic theories and models in consumer neuroscience research. In this section, we compare two platforms and identify potential benefits and limitations of these methods.

The first method is iMotions Platform (iMotions platform iMotions A/S, Kobenhavn V, Denmark). iMotions is an integrated analysis platform that measures consumer behavior and monitors different aspects of human responses to marketing stimuli using different sensors. iMotions platform provides full integration and synchronization support for more than 50 biosensors. iMotions can integrate up to six consumer neuroscience tools such as ET, facial expression analysis software, GSR, ECG and EEG headsets. iMotions also uses Application Programming Interface (API) and Lab Streaming Layerand for additional sensor integration. Most importantly, iMotions has a built-in survey tool that triangulates respondents' behavioral data (e.g., answers to survey) with neurophysiological responses, providing higher validity to consumer neuroscience experiments. iMotions allows users to perform studies in different environments such as laboratory, shops. This platform can be used to study consumer behavior and neurophysiological responses by exposing subjects to images, videos, games, apps, or software and allowing them to test real-like objects in product design studies and media/ad/website testing. It enables the easy set-up of participant groups, randomizations, and block designs as needed, and the data can be viewed both in real time and replay. iMotions software platform has been recently used in consumer neuroscience research. This was used to (1) measure willingness to pay, (2) the effect of music in advertisements, and (3) to evaluate the efficiency of online appointment booking platforms (Cuesta et al., 2018; Kulke et al., 2018; Ramsøy et al., 2018; Etzold et al., 2019). The main advantages of iMotion are that it can be adjusted to the needs of customers. There are different versions and the number of integrated tools can be changed. Another advantage of iMotions is that it can be used both in real-life or lab settings.

The second method is GRAIL (Gait Real-time Analysis Interactive Lab) system. The GRAIL system (Motek Medical BV, the Netherlands) is a new medical device that uses an instrumented dual-belt treadmill placed in a speed-matched and synchronized virtual reality (VR) environment and a motion-capture system (VICON system, Vicon Motion Systems Ltd UK). GRAIL allows users to perform clinical gait analysis, which consists of the computation of various gait parameters such as posture, muscle activation and ground reaction forces in real-time (Van den Bogert et al., 2013). The core element of the system is represented by the D-Flow software platform, which integrates both motion capture technology and a motion platform, allowing the subject to interact in real time with the system and receive feedback from it. D-Flow controls all the hardware connected to the system and also includes a 3D game engine to provide interactive VR environments to mimic real-life situations that engage subjects. In fact, it makes the subject part of a real-time feedback loop, in which multi-sensory input devices measure behavioral and physiological responses, while output devices return motor sensory, visual and auditory feedback to the subject. A semi-cylindrical 180°projection screen immerses the subject in a virtual reality environment that mimics real-life situations (i.e., a market). In addition to this, thanks to D-Flow software, it is possible to integrate other tools such as electromyography, electroencephalography, electrocardiography, galvanic skin response, eye tracking and head motions detection technology.

To our knowledge, GRAIL has been never used in consumer neuroscience research. However, it could represent a useful and complete tool for consumer neuroscience research for several reasons. D-Flow software has an intuitive interface that allows operators to easily control hardware, tailor applications or define their own applications. In addition, the D-Flow interface does not require programming skills, therefore, less training is required compared to other consumer neuroscience tools for researchers and practitioners who do not have technical skills (e.g., coding, programming). Using GRAIL would facilitate companies and universities to carry out consumer neuroscience experiments by reducing the time and expenses of training (e.g., fewer training sessions, in-house training). Finally, the use of fully customisable VR environments could increase the efficacy and the accuracy of consumer neuroscience research. Consumer neuroscience experiments too often simplify the complexity of the decision process because experiments are carried out in a laboratory setting (Lee et al., 2018; Alvino et al., 2019). Participants are subjected to a standardized procedure and there is no interaction with the external environment. The synchronized virtual reality (VR) environment of GRAIL immerses participants in a virtual scenario that reproduces real-life situations, while researchers can monitor participants' cognitive and behavioral phenomena using different types of tools. VR has been demonstrated to contribute to enhancing consumer experience and customer interactivity by directly impacting the users' sensory elements (Vrechopoulos et al., 2009; Bigne et al., 2016; Vickers, 1972).

GRAIL allows the recreation of real-life purchase situations, for instance, walking in a supermarket, mall or hotel while participants are still in a controlled environment. Additionally, GRAIL enables researchers to study brain activity and physiological responses while participants perform complex tasks (e.g., look at advertisement while walking) or make complex choices (e.g., choosing between several products on a shelf). VR offers the opportunity to develop completely new situations, impossible to create in the real world, and the development of contexts that will never be experienced by most people in real life.

Despite all the described advantages, it should be noted that the GRAIL system also has disadvantages. The first one is the costs of the system, which is around €400,000. These costs do not include other hardware to be integrated (e.g., EEG, EMG). The complete system needs a total space of at least 25 m^2^. The average time required for a experiment is about 45 min, including subject preparation, which is the phase that requires the most time, especially if the experimenter needs to compute gait parameters.

Compared to GRAIL, iMotions is less expensive. In fact, GRAIL consists of expensive integrated devices (treadmill, motion capture system, software, computers), while iMotions includes only the software platform and does not integrate by default any hardware. In addition, iMotions is highly portable, highly flexible and it allows also facial expression recognition. If used in a real world environment, iMotions could provide a higher user experience and interactivity than GRAIL. However, iMotions does not allow the measurement of gait parameters and it provides a limited user experience when used in a lab setting. In addition, it is not always possible to use iMotion in real-world situations due to logistic and economic reasons. In contrast, the GRAIL system can provide a consistent level of user experience and interactivity because of its ability to provide highly customisable VR environments.

GRAIL allows researchers to have a permanent lab in a small space mimicking real-life scenarios. Finally, the safe environment of GRAIL can help reduce motion artifacts that can be generated by moving in a wider environment. Table 5 summarizes the number of neuroscience tools that can be integrated and the advantages and disadvantages of both platforms.

**Table 5:** Advantages and disadvantages of modern platforms.

**Platform**	**Tools**	**Advantages**	**Disadvantages**
	Wearable EEG	Gait analysis	High cost
	Facial EMG	Custom. virtual env.	Not portable
GRAIL	ET	VR to improve user exp.	
	Head motion		
	ECG		
	GSR		
	Wearable EEG	Portable	No gait analysis
	ET	Relatively low cost	No custom. virtual env.
iMOTION	ECG		
	GSR		
	Head motion		
	Facial EMG		

## 7. Discussion and Conclusion

The application of physiological theories and neuroscience tools to marketing has rapidly grown in the last two decades. While the interest in consumer neuroscience is increasing, researchers in the field are facing new and complex challenges. Hence, it is important to have a clear overview of current (and potential) tools used in consumer neuroscience research. Thus, researchers (especially newcomers) might find it useful to identify or choose which tools are suitable to be applied in specific aspects of consumer neuroscience research. In this article, we examine present neuromarketing and consumer neuroscience literature with an aim to provide an overview of the use and characteristics of neuroscience tools used for studying consumer behavior.

We follow a literature review methodology as proposed by Webster and Watson (2002) in order to select relevant literature for our study. We evaluate a total of 219 publications to record details about the use and characteristics of consumer neuroscience tools. In section 2, we identify a wide range of neuroscience tools described as consumer neuroscience research (i.e., 15 tools). However, the findings of our study highlighted that the number of tools used in consumer neuroscience research might be lower. We identify seven tools that have been practically used in the field, namely EEG, fMRI, fNIRS, ECG, ET, GSR, and facial expression recognition software. We do not find any empirical evidence for the use of the other tools. We propose a criterion to classify the practically used consumer neuroscience tools as shown in Figure 4. We believe that classifications including self-reports and questionnaires, like Ramsøy (2015) give a more accurate and realistic idea of the variety of tools that can be used in consumer neuroscience research. In our classification, we divided consumer neuroscience tools into three categories, namely (1) behavioral (self-reports, observations, survey), (2) physiological (ECG, ET, ECG, fERS), and (3) neurophysiological (electrical: EEG and metabolic: fMRI, fNRIS).

We also evaluate the popularity of the consumer neuroscience tools based on how often they were used by researchers. Studies have argued that fMRI is the most used neuroimaging tool in consumer neuroscience research (Smidts et al., 2014; Ramsøy, 2015; Harris et al., 2018). However, we find that EEG, both traditional and wearable, is the most popular tool, followed by Eye Tracking. These results are also in line with a survey conducted by the Neuromarketing Science and Business Association (NMSBA) on consumer neuroscience vendors in 2018 (Cherubino et al., 2019). This suggests that there has been a change in the use and applications of consumer neuroscience tools in the last 5 years. It is possible that researchers no longer use fMRI because there are other tools that are available for consumer neuroscience research. It might also be possible that researchers are opting for more portable, less invasive and low-cost tools. Although fMRI is not considered an invasive tool, subjects might suffer inconvenience due to being in a small space, an inability to move and excessive noise during the scan. Also, fMRI applications in consumer neuroscience are also limited due to disturbing environmental factors (e.g., lightning conditions, auditory noise). Wearable EEG, fNIRS, ET, GSR, ECG, and Facial Expression recognition software allow higher flexibility to the subjects (e.g., sit, walk). Thus, wearable tools allow the designing of field experiments and not only laboratory experiments. Thus, these tools can help recreate a natural setting for consumer neuroscience experiments compared to fMRI. Additionally, some studies have implied that all neuroscience tools can be used in consumer neuroscience research. We argue that some neuroscience tools are not suitable for marketing research due to moral and ethical implications. In particular, we suggest that tools such as PET, TMS, and SPET should not be used in consumer neuroscience research. In fact, PET, TMS, and SPET may be too invasive or harmful to be used in consumer neuroscience studies. These tools expose participants to unnecessary risks, such as pain, fainting, seizures, and injection of radiopharmaceuticals.

In section 4, we analyzed the advantages, disadvantages, average costs and time preparation for each of these tools. In total, we identified three neurophysiological tools (EEG, fMRI, and fNIRS) and four physiological tools (ET, GSR, ECT, fERS). Our findings suggest that neurophysiological tools are usually more expensive, time consuming and technical knowledge-based (e.g., coding) compared to physiological tools. Similarly, neurophysiological tools can only be used in lab settings, except for the wearable EEG. In contrast, physiological tools are low-cost, easy to integrate with other tools and do not require much technical-knowledge and preparation time. Furthermore, wearable physiological tools can be used outside lab settings such as supermarkets and hotels. Thus, they contribute to study consumer behavior in real-life scenarios.

To understand how these tools improve marketing research we also analyzed the cognitive processes, behavioral measurements, marketing applications and types of product for each consumer neuroscience tool (e.g., EEG, ET). Our findings show that neurophysiological tools allow the study of cognitive processes (e.g., attention, excitement) and behavioral measurement for a wide range of marketing issues. In contrast, physiological tools such as ECG, GSR, and fERS have limited applications in marketing as they only measure a few cognitive processes (arousal, attention, engagement, emotion, and valence). We found that consumer neuroscience tools are currently used to improve marketing strategies for advertising, branding, product experience, online experience, product development and pricing. In particular, we found that the most important contribution consumer neuroscience tools can offer to marketing is the study of consumer behavior in advertising. Consumer neuroscience research focusses on important aspects of advertisement strategies, such as advertisement effectiveness (pre- and post-testing) and target audience selection (e.g., young, adults, male, female). Based on our findings, a small number of studies used neuroscience tools to study product experience. It suggests that research in this specific marketing issues is still in an initial phase. However, there are new and promising applications of consumer neuroscience tools to marketing research. Our findings suggest that consumer neuroscience tools contribute to both theoretical and practical aspects of marketing research.

In recent years, we have seen an increase in the number of studies that integrate two or more consumer neuroscience tools. We believe that ready-to-use platforms might help researchers build more realistic theories and models in consumer neuroscience research. In fact, platforms can contribute to (1) reducing time and cost to set up experiments, (2) more reliable data processing, (3) setting up large scale studies, and (4) collecting simultaneous information on physiological and neurophysiological processes. In this study, we identified two ready-to-use platforms that integrate some of the consumer neuroscience tools discussed in this research, namely iMotions and GRAIL. The first platform, iMotions, has been previously used in consumer neuroscience studies to investigate willingness to pay and consumer satisfaction for different products (e.g., bags, clothes, FMCG, shoes) and services (e.g., booking platform). To the best of our knowledge, this is the first study to consider GRAIL (the second platform identified by us) for use in consumer neuroscience research.

Our findings show that both platforms can integrate six tools and measure both physiological and neurophysiological processes. In particular, iMotion can integrate wearable EEG, facial electromyography, ET, ECG, GSR, head motion. GRAIL integrates wearable EEG, facial electromyography, ET, ECG, GSR and head motion. GRAIL can also offer gait analysis. Compared to GRAIL, iMotion is relatively low cost and portable. Thus, iMotion can also be used outside lab settings. However, GRAIL incorporates synchronized virtual reality (VR) that can easily reproduce real-life situations. GRAIL's customizable VR allows for the recreation of different environment in one experiment. This platform can help the improvement of consumer experience and customer interactivity in consumer neuroscience studies.

We believe that this study provides a comprehensive overview of the consumer neuroscience tools that have been practically used by researchers. We hope that this work will help researchers and practitioners in choosing the correct tool for their experiment by providing them with necessary information to evaluate the pros and cons of available tools. We also emphasize the potential of upcoming ready-to-use platforms that can help make consumer neuroscience experiments reliable, quick, and low cost.

## 8. Limitations and Future Work

This study comes with some limitations that offer opportunities for future research. Although we tried to minimize methodological shortcomings, the present study is not completely bias-free. In this study, we consider publications in the English language only; there is a possibility that we have overlooked tools whose practical use has been explained only in non-English publications. Also, we have focussed on only a few aspects of the moral and ethical implications of the use of consumer neuroscience tools. For future work, we suggest researchers analyse the effect of consumer neuroscience research on personal privacy and ethical values and principles. We would like to invite researchers to investigate the possible contributions of neuroscience tools to others disciplines such as economics and organizational behavior.

For a successful consumer neuroscience experiment, it is also important for researchers to design the experiment well (Fink et al., 2007; Murray and Antonakis, 2019). In this review, our goal was to describe consumer neuroscience tools, hence, we do not look at the experimental design aspect of consumer neuroscience within the scope of this review. We would like to investigate the success and failure of experimental designs in consumer neuroscience research in the future.

## 9. Summary

- Consumer neuroscience tools can be divided in three categories based on the type of measurements: (1) Self reports and behavioral, (2) Physiological and (3) Neurophysiological.
- The tools currently used in consumer neuroscience research are EEG, fMRI, fNIRS, ECG, ET, GSR, and fERS. EEG is the most commonly used tool in consumer neuroscience research.
- Physiological tools are usually cheaper and portable compared to neurophysiological ones.
- Consumer neuroscience tools have applications in several marketing domains such as advertising, branding, online experience, pricing, product development and product experience.
- Ready-to-use platforms (iMotions and GRAIL) measure behavioral, physiological, and neurophysiological responses simultaneously. GRAIL includes a personalized virtual reality setting that enhances the consumer experience and customer interactivity.

## Author Contributions

All authors of this article had a significant contribution in its preparation. HR's inputs were critical in research design and formulation of arguments. LA, LP, and AA were involved in reading of literature, writing, and design of figures and tables. LA specialized in neuromarketing. LP was an expert on neuroscience tools. AA specialized in technology and information systems. HR was a specialist on Marketing.

## Conflict of Interest

The authors declare that the research was conducted in the absence of any commercial or financial relationships that could be construed as a potential conflict of interest.

## Footnotes

^1^(2020). *Marketing and Consumer Research*. Available online at: https://www.tobiipro.com. Emotion Research Lab.

^2^(2020). *Facial Action Coding System*. Available online at: https://emotionresearchlab.com. Emotion Research Lab.
