# Corrigendum: Picking your brains: where and how neuroscience tools can enhance marketing research

**DOI:** 10.3389/fnins.2024.1426471

**Published:** 2024-05-16

**Authors:** Letizia Alvino, Luigi Pavone, Abhishta Abhishta, Henry Robben

**Affiliations:** ^1^Center for Marketing and Supply Chain Management, Nyenrode Business University, Breuklen, Netherlands; ^2^Neuromed, Mediterranean Neurological Institute, Isernia, Italy; ^3^Hightech Business and Entrepreneurship Group (HBE), University of Twente, Enschede, Netherlands

**Keywords:** neuromarketing, consumer neuroscience, review, iMotion, GRAIL, marketing, neurophysiological tools, physiological tools

In the published article, the reference for “Vickers (2017)” was incorrectly inserted as “Vickers, N. J. (2017). Animal communication: when I'm calling you, will you answer too? Curr. Biol. 27, R713–R715. 10.1016/j.cub.2017.05.064 The correct reference should be:

“Vickers, D. L. (1972). *Sorcerer's Apprentice: Head-Mounted Display and Wand* (Ph.D. Thesis). The University of Utah, Salt Lake City, UT, United States.”

In the published article, there was also an error in “*4.5. Eye Tracking*,” paragraph 2 regarding the reported equipment costs. Costs were reported to range between “€100,000 and 300,000” but this should be “€100 and 30,000.” The corrected paragraph appears below:

“ET has been widely used in consumer neuroscience research to study visual behavior (e.g., fixation, gaze, pupil dilatation), customers' visual attention mechanisms and consumers' engagement (Zamani et al., 2016; Ungureanu et al., 2017). ET has several advantages: it is portable, non-invasive, simple to use and relatively inexpensive. ET has a cost ranging between €100 and 30,000 euros, depending on the level of the technology and whether the software to acquire and analyse data is included^1^. An ET experiment needs only a technician and, eventually, a data analyst. The average time needed to perform an ET experiment is about 15 min since the subject set-up is very fast. This time covers only the time needed to perform the experiment by the subject. Not all the ET has a high flexibility as some ET models might not work efficiently with glasses and contact lenses. ET is also characterized by a high level of integration with other tools due to its portability and because it is a “ready-to-use” device. To have more reliable results, ET should be used in combination with other tools.”

The authors apologize for these errors and state that this does not change the scientific conclusions of the article in any way. The original article has been updated.

